# Association Between Resident Duty Hours and Self-study Time Among Postgraduate Medical Residents in Japan

**DOI:** 10.1001/jamanetworkopen.2021.0782

**Published:** 2021-03-05

**Authors:** Kazuya Nagasaki, Yuji Nishizaki, Tomohiro Shinozaki, Hiroyuki Kobayashi, Yasuharu Tokuda

**Affiliations:** 1 Department of Internal Medicine, Mito Kyodo General Hospital, University of Tsukuba, Ibaraki, Japan; 2 Department of Medical Education, Juntendo University School of Medicine, Tokyo, Japan; 3 Department of Information and Computer Technology, Faculty of Engineering, Tokyo University of Science, Tokyo, Japan; 4 Muribushi Okinawa for Teaching Hospitals, Okinawa, Japan

## Abstract

This cross-sectional study uses information from the General Medicine In-Training Examination survey in Japan to assess the association between resident duty hours and self-study time.

## Introduction

Sufficient self-study time (SST) for postgraduate residents is important for their professional development.^1,2^ Self-study time has a positive linear association with the acquisition of clinical knowledge.^3^ In 2024, duty-hour (DH) restrictions will be implemented in Japan for postgraduate residents; therefore, the association of DHs with resident study habits must be evaluated.^4^ In the US, DH restrictions have had minimal or no association with resident SST.^5^ The objective of this study was to assess the association between DHs and resident SST using information from the General Medicine In-Training Examination (GM-ITE) survey in Japan.

## Methods

We conducted a cross-sectional survey among 5590 postgraduate residents across Japan who had taken the GM-ITE from January 20 to 26, 2020.^6^ This examination evaluates the acquisition of clinical knowledge. Participants were asked about mean DHs and SST, as well covariates including sex, postgraduate year, the number of emergency department duties, and the mean number of inpatients in their care. All participants signed a consent form. The Ethics Review Board of Mito Kyodo General Hospital approved the study. This study followed the Strengthening the Reporting of Observational Studies in Epidemiology (STROBE) reporting guideline.

The dependent variable was SST per day, which comprised 5 categories (none, 1-30, 31-60, 61-90, and ≥91 minutes). The independent variable was DHs per week. This variable was divided into 8 categories as follows: category 1 (<45 hours), category 2 (≥45 to <50 hours), category 3 (≥50 to <55 hours), category 4 (≥55 to <60 hours), category 5 (≥60 to <65 hours), category 6 (≥65 to <70 hours), category 7 (≥70 to <80 hours), and category 8 (≥80 hours).

We examined the association between resident SST and DHs using a proportional odds regression analysis (ie, an ordered logistic model with a cumulative logit link) with adjustment. Category 5 was set as a reference. We excluded participants from the analysis who did not provide responses for SST or DHs. Hospital variability was accounted for by cluster-robust variance using generalized estimating equations. All analyses were conducted using SAS, version 9.4 (SAS Institute Inc). All *P* values are 2-sided without setting significance level to show compatibility between data and null association under the models.

## Results

Of 6164 residents, 5590 at 528 hospitals were included in the analysis. Of the included residents, 2837 (50.8%) were in their first postgraduate year and 1769 (31.6%) were women. Their SSTs were as follows: 1 to 30 minutes, 2067 residents (37.0%); 31 to 60 minutes, 2330 residents (41.7%); 61 to 90 minutes, 788 residents (14.1%); 91 minutes or longer, 166 residents (3.0%); and none, 239 residents (4.3%). They reported their DHs as follows: category 1, 133 residents (2.4%); category 2, 514 residents (9.2%); category 3, 827 residents (14.8%); category 4, 832 residents (14.9%); category 5, 1047 residents (18.7%); category 6, 754 residents (13.5%); category 7, 676 residents (12.1%); and category 8, 807 residents (14.4%). The Table shows the distribution of SST categories in each DH category.

**Table.** Proportion of Self-study Time Categories in Each Duty-Hour Category of 5590 Participants

Self-study time, min/d	Total, No. (%) (N = 5590)	Categorized by duty hours, No. (%)^a^							
		Category 1 (n = 133)	Category 2 (n = 514)	Category 3 (n = 827)	Category 4 (n = 832)	Category 5 (n = 1047)	Category 6 (n = 754)	Category 7 (n = 676)	Category 8 (n = 807)
None	239 (4.3)	10 (7.5)	17 (3.3)	28 (3.4)	35 (4.2)	36 (3.4)	29 (3.8)	29 (4.3)	55 (6.8)
1-30	2067 (37.0)	73 (54.9)	253 (49.2)	323 (39.1)	314 (37.7)	374 (35.7)	245 (32.5)	214 (31.7)	271 (33.6)
31-60	2330 (41.7)	33 (24.8)	196 (38.1)	352 (42.6)	353 (42.4)	435 (41.5)	360 (47.7)	294 (43.5)	307 (38.0)
61-90	788 (14.1)	14 (10.5)	43 (8.4)	104 (12.6)	107 (12.9)	170 (16.2)	100 (13.3)	112 (16.6)	138 (17.1)
≥91	166 (3.0)	3 (2.3)	5 (1.0)	20 (2.4)	23 (2.8)	32 (3.1)	20 (2.7)	27 (4.0)	36 (4.5)

^a^ Resident duty hours per week comprised 8 categories: category 1 (<45 hours), category 2 (≥45 to <50 hours), category 3 (≥50 to <55 hours), category 4 (≥55 to <60 hours), category 5 (≥60 to <65 hours), category 6 (≥65 to <70 hours), category 7 (≥70 to <80 hours), and category 8 (≥80 hours).

After adjustment for sex, postgraduate year, the number of emergency department duties, and the number of inpatients in their care, the resident SST was shorter for categories 1 to 4 than for category 5 (Figure). In contrast, we observed little or no differences in SST between categories 6 to 8 and category 5.

**Figure.**
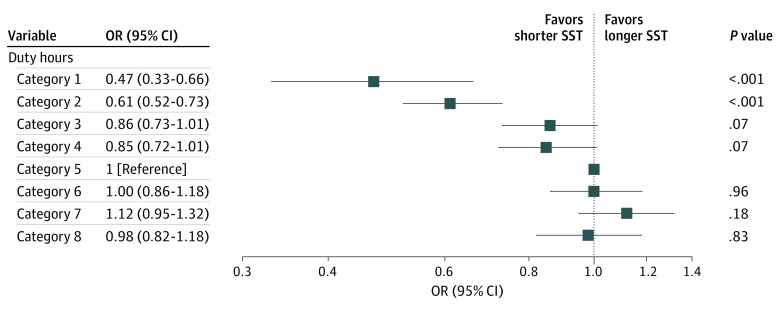
Association Between Resident Duty Hours and Self-study Time (SST) of 5590 Participants Resident duty hours per week comprised the following 8 categories: category 1 (<45 hours), category 2 (≥45 to <50 hours), category 3 (≥50 to <55 hours), category 4 (≥55 to <60 hours), category 5 (≥60 to <65 hours), category 6 (≥65 to <70 hours), category 7 (≥70 to <80 hours), and category 8 (≥80 hours), adjusted for sex, postgraduate year, the numbers of monthly emergency department duties, and mean number of inpatients in residents’ care. Category 5 was set as a reference. OR indicates odds ratio.

## Discussion

In this study, postgraduate residents with DHs less than 60 hours per week had a shorter SST than those with DHs between 60 and 65 hours per week. This result indicated that extra time during residency may not necessarily be associated with more SST. It is possible that less-motivated residents may have chosen hospitals with shorter DHs. Also, the DH restrictions in Japan may run the risk of shortening resident SST. Providing online educational resources and conducive learning environments in hospitals may improve resident study habits.^5^

Our study had the potential for selection bias. Because the GM-ITE is voluntary for training hospitals, approximately one-third of residents in Japan participated in this study. In 2024, when DH restrictions begin in Japan, each residency program must encourage residents to secure more SST to maintain resident quality.
